# Evaluation of the quality of Primary Health Care services for children: reflections on the feasibility of using the Brazilian version of the Primary Care Assessment Tool as a routine assessment tool

**DOI:** 10.31744/einstein_journal/2019AO4333

**Published:** 2019-02-01

**Authors:** Liz Ponnet, Sara Willems, Veerle Vyncke, Aylene Emilia Moraes Bousquat, Ana Luiza d’Ávila Viana, Guilherme Arantes Mello, Marcelo Demarzo

**Affiliations:** 1 Universidade Federal de São Paulo, São Paulo, SP, Brazil.; 2 Ghent University, Ghent, Belgium.; 3 Universidade de São Paulo, São Paulo, SP, Brazil.

**Keywords:** Quality of health care, Primary health care, Child, Brazil, Qualidade da assistência à saúde, Atenção primária à saúde, Criança, Brasil

## Abstract

**Objective:**

To assess the quality of the Primary Health Care services provided to children and the feasibility of using the Brazilian version of Primary Care Assessment Tool (PCAT-Brazil) as a routine quality assessment tool.

**Methods:**

A cross-sectional study was carried out in Joanópolis, a small rural town in the State of São Paulo (SP), Brazil. Seven health professionals and 502 caretakers of children using the public health center were interviewed using the PCAT-Brazil, collecting data on the core and related attributes of Primary Health Care provided to children. The score of each attribute was calculated.

**Results:**

Caretakers rated as good the following attributes; “degree of affiliation”, “first contact care − use of services”, “coordinated care”, and “comprehensive care − available services”. The attributes of “first contact accessibility”, “long term person care”, “comprehensive care − offered services” and “family- and community-oriented care” were scored as poor. The health professionals only rated the attribute of “first contact accessibility” as satisfactory, and considered that all other Primary Health Care attributes needed improvement. To conduct this study, at least 1,241 working hours were invested, and the estimated budget was R$12.900,00 (or U$3,953.00).

**Conclusion:**

The use of the PCAT-Brazil as a routine assessment and planning tool seemed to be not feasible in the given setting due to high costs, lack of trained personnel and the huge workload. To overcome the encountered obstacles, advices are given based on field experience.

## RESUMO

**Objetivo:**

Avaliar a qualidade dos serviços de Atenção Primária à Saúde prestados às crianças e a viabilidade de usar a versão brasileira da *Primary Care Assessment Tool* (PCAT-Brazil) como ferramenta rotineira de avaliação da qualidade.

**Métodos:**

Estudo transversal, realizado em Joanópolis, um pequeno município rural no Estado de São Paulo. Sete profissionais de saúde e 502 responsáveis legais de crianças que usaram o centro de saúde foram entrevistados usando a PCAT-Brazil, para coleta de dados sobre os atributos essenciais e derivados dos serviços de Atenção Primária à Saúde oferecidos às crianças. Foi calculado o escore para cada atributo.

**Resultados:**

Os responsáveis classificaram como satisfatórios os atributos como “grau de afiliação”, “acesso de primeiro contato − utilização”, “coordenação” e “integralidade − serviços disponíveis”. Os atributos “acesso de primeiro contato − acessibilidade”, “longitudinalidade”, “integralidade − serviços prestados” e “orientação familiar e comunitária” foram classificados como insatisfatórios. Por outro lado, os profissionais de saúde apenas classificaram o atributo “acessibilidade do primeiro contato” como satisfatório e consideraram todos os outros atributos de Atenção Primária à Saúde como necessidade de melhoria. Para realizar este estudo, foram investidas pelo menos 1.241 horas de trabalho e o orçamento estimado foi de R$12.900,00 (ou U$3,953.00).

**Conclusão:**

Usar a PCAT-Brazil como ferramenta de avaliação e planejamento de rotina pareceu não ser viável neste estudo, pelos altos custos, pela falta de pessoal treinado e pelo grande número de horas de trabalho exigido. Para superar os obstáculos encontrados, conselhos foram formulados com base na experiência de campo.

## INTRODUCTION

Over the last 20 years Brazil, a country traditionally characterized by regional, socioeconomic and health care inequalities,^(^ ^1^ ^)^ has made progress towards provision of more equitable health care.^(^ ^2^ ^)^ A milestone in this progress has been the creation of the national Unified Health System (SUS - *Sistema Único de Saúde)* , that establishes the right of universal and free access to integrated health care services for all Brazilian citizens.^(^ ^3^ ^)^ Primary Health Care (PHC) services are the preferential gateway to the national health system,^(^ ^4^ ^)^ a policy shown to reduce health care inequities.^(^ ^5^ ^)^ When a Brazilian citizen feels the need to see a doctor, he can visit a local public PHC center where he will be assisted by a nurse, a clinician (adult user), a pediatrician (child user) or a gynecologist (female user). This is considered the traditional model of PHC, still serving 38% of the Brazilian population.^(^ ^6^ ^)^ The majority of Brazilians (62%) are now subscribed to the Family Health Strategy (FHS) model, in which a multidisciplinary team of a family doctor, a nurse, an nurse technician, four community healthcare workers and an oral health care team are responsible for a defined population in a delineated geographical area.^(^ ^7^ ^)^ In this model, a community health worker will visit each family once a month, and will constitute the link between the community and the FHS unit for all health-related issues. If a SUS’ user expresses the need to make a medical appointment, he will do so at his usual FHS unit, where his known family doctor will provide care to most common health problems, and he will be referred to other specialists if the family doctor judges this to be necessary. Besides medical care, a user can rely on other PHC-services such as vaccinations, wound dressings, dental care, as well as health promotion activities.

Each city is responsible for organizing its own public PHC services,^(^ ^4^ ^)^ managed by the municipal health authorities, with active participation of the users of SUS. Over time, cities have been moving from the traditional PHC model to the FHS-model, supported by strong evidence that the latter model reduces health care inequities and infant mortality rates.^(^ ^2^ ^,^ ^8^ ^,^ ^9^ ^)^

Brazil counts 5,570 cities; 70% of these cities are small towns with less than 20.000 inhabitants.^(^ ^10^ ^)^ A huge variety of locally adapted PHC models can be found, especially in rural or remote mountain areas, where it can be challenging to guarantee access to PHC services near to the people’s homes, due to long distances to the health care unit, non-paved roads that are inaccessible during the rainy season, and even the lack of communication means such as mobile telephone networks.

Primary Health Care provides an answer to these barriers by its nature. The core attributes^(^ ^11^ ^)^ of PHC can be defined as: first-contact accessibility and use of care, meaning that a person consults the PHC unit when a new health need arises; long-term patient care, meaning that a person is followed over time at his regular health center or FHS unit for whatever health-related problem; comprehensive care, meaning that the provided care includes preventive and curative care, including referrals to relevant specialist care providers if needed; and coordination of care, entitling the PHC unit as a key partner in the management of the care provided to the patient by different care providers by keeping and a good user-health professional relationship.

Primary Health Care is further defined by the following three attributes:^(^ ^11^ ^)^ (1) family-centered care, referring to the understanding of the complex influences of the social context on an individual’s health need; (2) community-oriented care, meaning the PHC unit recognizes the (unmet) health needs of the community of which the user is part and understands the health-related characteristics of this particular community; and (3) cultural competence, meaning that care is provided in a way that is in line with the culture and reality of the patient in order to ensure that care is accessible and acceptable for the patient.

Various instruments have been developed to evaluate the attributes of PHC, including the Primary Care Assessment Tool (PCAT).^(^ ^12^ ^)^ The PCAT has been translated and adapted to be used in different countries,^(^ ^13^ ^-^ ^15^ ^)^ including Latin American countries such as Uruguay,^(^ ^16^ ^)^ Argentina^(^ ^17^ ^)^ and Brazil.^(^ ^18^ ^)^ This reflects the main advantage of the PCAT, being its cross-cultural reliability. The Brazilian Ministry of Health encourages the use of the (PCAT-Brazil) for evaluating and monitoring the quality of PHC services.^(^ ^19^ ^)^ Many national studies have been performed to evaluate the PHC attributes using the PCAT. Most of them however were performed in large cities, with some studies being part of a Ministry of Health-World Bank funded research project on the Expansion and Consolidation of the Family Health Strategy (PROESF) in cities with over 100.000 inhabitants.^(^ ^20^ ^,^ ^21^ ^)^

## OBJECTIVE

Assess the quality of the Primary Health Care services provided to children in a small rural town and to evaluate the feasibility of using the Brazilian version of Primary Care Assessment Tool (PCAT-Brazil) as a routine quality assessment tool.

## METHODS

### Setting

The study was conducted in Joanópolis, a small rural town of 12,610 inhabitants, located in the Mantiqueira Mountains in the State of São Paulo.^(^ ^10^ ^)^ Poverty incidence in the area is high, with 31.30% of the population in the town earning a monthly income up to half a Brazilian minimum wage.^(^ ^10^ ^)^ Overall monthly *per capita* income is R$607,00 (US$190.7), equivalent to 1.2 Brazilian minimum wage.^(^ ^10^ ^)^ The majority of the population older than 25 years (76.3.%) has less than 8 years of schooling.^(^ ^10^ ^)^ Half of the population lives in the center of the town and half in the rural mountain areas. The territory is extensive (374.28km^2^),^(^ ^10^ ^)^ with some people living more than 30km away from the center of the town, where all health care services are concentrated. There is no public transportation available. The town counts one public health center offering PHC services, one emergency room providing emergency care 24 hours, ran by a philanthropic organization, and one private practitioner physician office. The coefficient of medical doctors attending in the public sector is 0.72 per 1,000 inhabitants.^(^ ^22^ ^)^ The health professionals working at the health center are two nurses, three nurse technicians (2.2 full time equivalents − FTE), three clinicians (2FTE), two pediatricians (1FTE), two gynecologists (1FTE), and other specialists such as an orthopedic surgeon (1/6FTE), a psychiatrist (1/5FTE) and a cardiologist (1/5FTE). There is sporadic provision of medical outreach activities to the rural areas.^(^ ^22^ ^)^ The Family Health Strategy, with a multidisciplinary team attending a defined population, was not yet been put into practice at the time of the study.^(^ ^22^ ^)^ The majority of the population (79.9%) does not have any private health insurance plan and relies exclusively on the public health services.^(^ ^22^ ^)^

### Ethical considerations

This study received approval of the Ethical Committee of the *Universidade Federal de São Paulo* , under the Brazilian number CAAE: 02244812.0.0000.5505, as well as approval of the Ethical Committee of the Ghent University in Ghent, Belgium, under the number BE670201420498.

### Design

This is a cross-sectional study applying the PCAT-Brazil to child-users and to health professionals of the public health center in Brazil.

### Assessment tool

The PCAT-Brazil child version is the tool adapted to the Brazilian reality,^(^ ^19^ ^)^ which consists of a consumer version (questionnaire applied to caretakers of child-users) and a professional version (survey for health professionals). The consumer version of the PCAT-Brazil child version has 55 items, while the professional version consists of 77 items. These questionnaires measure the degree of affiliation to a PHC-unit, the use of these health care services, and the PHC attributes. The four main PHC attributes are “first-contact” care, long-term person care, coordination of care (including integrated care and information systems), and comprehensive care of available and provided services. The two related PHC attributes are family- and community-oriented care. For each attribute, a score can be calculated on a scale ranging from zero to 10. The sum of all attributes, calculated following predetermined rules,^(^ ^19^ ^)^ results in the general PHC score, which expresses the overall quality of provided PHC services. The essential PHC score can also be calculated based on only the four main attributes, and then reflects the performance of the core domains of the offered PHC services.

### PCATool-Brazil child version: questionnaire of caretakers

#### Subjects: selection of the respondents

Eligible participants were parents or legal representatives (further called “caretaker”) of children aged zero to 12, entering the public health center and seeking non-urgent medical care for their child. The caretaker was approached in the waiting room and invited to participate in the study. After reading a letter explaining the study and signing the Informed Consent form, the PCAT-Brazil child version was administered orally.

Exclusion criteria considered caretakers with mental disabilities. In case of a caretaker seeking care for two or more children, the questionnaire was applied to the youngest child only.

#### Sample

The sample size of caretakers of child-users was estimated at n=319. We adopted a confidence interval of 95% and a 5% confidence level, considering the population of 1,861 children aged zero to 12 years who were resident in Joanópolis, in 2012.

#### Data collection

Of the 508 caretakers who were approached to participate in the study, 6 refused (response rate was 98.9%). Data from 502 caretakers were collected using the PCAT-Brazil child version between October 2013 and August 2014.

#### Outcome variables and measures

The scores for each attribute, the general and essential PHC scores, are the outcome variables.

The higher the score, the better, with 6.6 being the cut-off point for high quality care.^(^ ^18^ ^)^

#### Data analysis

Data were entered in Excel by one researcher and double-checked by a second researcher. Statistical Package for Social Sciences (SPSS) version 23.0 for Windows was used for data processing. Results are shown as mean scores and their 95% confidence interval.

## PCATool-Brazil health professional version

### Subjects

All professionals working in the public PHC service in Joanópolis who assisted children, as well as two local health managers, were invited to participate in the study (n=8). All but one agreed to participate. One of the professionals did not answer the PCAT-Brazil due to recent employment at the health center, bringing the total number of respondents of PCAT-Brazil professional version to 7. All included health care professionals held a university degree (medical doctors and registered nurses).

### Outcome variables and measures

The PCAT-Brazil professional version allows calculating scores for each attribute, as well as the general and essential PHC score, indicating the performance of the PHC services from the health professional’s point of view.

The numeric scores range from zero to 10, with 6.6 being the cut-off point for well performing PHC services.^(^ ^18^ ^)^

### Data collection

After provision of consent, the health professional filled in the professional version of the PCAT-Brazil and sent it back to the researcher.

### Data analysis

Data were entered in Excel by one of the researchers and revised by another researcher. Statistical Package for Social Sciences version 23.0 for Windows was used for data processing. Results are shown as mean scores along with their 95% confidence interval.

## PCATool-Brazil health professional version

To assess the feasibility of the PCAT-Brazil study, the total man-hours, budget and timeline were recorded, as well as enabling and disabling factors described by the main researcher.

## RESULTS

### Evaluation of the quality of Primary Health Care services provided to children from the caretaker’s point of view

The caretakers evaluated the overall quality of PHC-services provided to their children as unsatisfactory: the general PHC score is 5.62. However, if only the core domains of PHC are considered, the parents attribute a better score: the essential PHC score is 6.92.

Most caretakers considered the health center as the place where they usually took their child for a health need: the degree of affiliation to the health center was 7.96. They also used the health center often as the first contact care, which was scored as 9.57. The accessibility was considered low (4.09), as well as the long-term person care (5.48). The coordination of care (8.54) and the information systems (7.58) were considered satisfactory from the caretakers’ point of view. The comprehensive care attribute was positive for the component of available services (7.20), and almost positive for the offered service (6.23). Family-orientated care scored low (2.04) and community-oriented care was almost absent (0.01). Table 1 shows the mean scores for attributes with a 95% confidence interval, based on the experience of child users.

**Table 1:** Primary Health Care (PHC) attributes, mean scores and 95% confidence interval (CI95%) for child users

PHC-attributes	n	Score	CI95%
Degree of affiliation	502	7.96	7.77-8.15
First contact care			
Use of services	502	9.57	9.46-9.69
Accessibility	502	4.09	3.93-4.26
Long term person care	502	5.48	5.39-5.58
Coordinated care/integrated services	100	8.54	7.88-9.20
Coordinated care/information systems	502	7.58	7.44-7.73
Comprehensive care/available services	448	7.20	7.09-7.32
Comprehensive care/offered services	495	6.23	5.89-6.57
Essential PHC-score		6.92	6.82-7.01
Family oriented care	500	2.04	1.83-2.26
Community oriented care	502	0.01	-0.01-0.02
General PHC-score		5.62	5.53-5.70

### Evaluation of the quality of Primary Health Care services provided to children from the health professionals’ point of view

The health professionals evaluated the PHC services provided to children as unsatisfactory (cut-off <6.6). The general score PHC-score was 5.52. Even if only the main PHC attributes were considered, the essential PHC score still would be negative (5.67). First contact accessibility scored well (7.20). Long term person care (5.57), coordination of care (5.32), coordination of information systems (4.44), comprehensive care available service (5.95) and offered services (5.56) were considered negative. Family- (5.71) and community-oriented care (4.44) scored low. Table 2 summarizes the attribute, general and essential PHC scores from the health professionals’ point of view.

**Table 2:** Primary Health Care (PHC) attributes according to Primary Care Assessment Tool, professional version

PHC-attributes	Score (n=7)
First contact care. Accessibility	7.20
Long term person care	5.57
Coordination of care/integrated services	5.32
Coordination of care/information systems	4.44
Comprehensive care/available services	5.95
Comprehensive/offered services	5.56
Essential PHC-score	5.67
Family oriented care	5.71
Community oriented care	4.44
General PHC-score	5.52

### Feasibility of using Primary Care Assessment Tool-Brazil as a routine assessment tool

To conduct this study, at least 1,241 working hours were invested, of which 39% in the design of the study, 21% in data collection, 13% in data analysis, writing the report and diffusing the preliminary results. The study started in 2012 and results were disseminated in March 2016. The estimated budget was R$12.900,00 (equivalent to US$3,953.73). Table 3 summarizes the invested man-hours, budget and timeline of the PCAT-Brazil conducted in Joanópolis.

**Table 3:** Invested man-hours, budget and timeline of the Primary Care Assessment Tool-Brazil (PCAT-Brazil)

	Man hours (hours)	Budget (R$)	Timeline	

			Starting date	Final date
Writing study protocol	480	0	Apr 4, 2012	July 30, 2012
Obtaining ethical approval	48	0	July 30, 2012	Feb 18, 2013
Obtaining approval from local health authorities	30	0	Ago 1, 2012	July 1, 2013
Informing health professionals and staff health center	24	0	June 1, 2013	July 20,2013
Preparing data collection (copying tools and consents)	8	1.800,00	June 1, 2013	July 25,2013
Training of interviewers	56	0	July 1, 2013	July 30, 2013
Collecting data PCAT-Brazil	265	5.100,00	July 25, 2013	Ago 11, 2014
Transportation and communication		1.000,00		
Input data (double-check)	160	4.000,00	May 1, 2014	Dec 1, 2014
Analyzing data	40	0	Jan 5, 2015	Jan 11, 2015
Writing up results	50	0	Jan 12, 2015	Feb 5, 2015
Diffusing the results	80	1.000,00	Jan 12, 2015	Ongoing
Total	1,241	12.900,00		

Motivation of health care personnel and support of the local health manager were considered as enabling factors to conducting the PCAT study in this particular context.

Although the Brazilian Ministry of Health provided the PCATool-Brazil online and stresses the importance of using the tool as an instrument to measure the quality of the PHC services on a routine basis, the researchers encountered some difficulties. Firstly, the Ministry of Health did not foresee funding for such studies, and even a small scale study as the present one, this kind of study had a reasonable cost. Secondly, the Ministry of Health provided the tool, but not the program to calculate the scores. Indeed, calculating the scores was doable, but time consuming and a constant quality check was needed. Other authors^(^ ^23^ ^)^ also pointed out that a shorter version of the PCAT-Brazil would be helpful to enable using the tool as a routine assessment. In addition, performing the research in a rural area was challenging because of communication difficulties (poor or no internet connections or even energy black outs during the rainy season), long distance to university or research centers, lack of public transportation etc. Also, practical problems had to be solved, such as the small space in a health center that had to be shared by researchers and health staff. During the study, there was a turnover of a local health manager because of changing in political administration; this unexpected change was considered a disabling factor, challenging the continuity of the research project.

## DISCUSSION

### Evaluation of the quality of Primary Health Care services provided to children in this rural town

#### General and essential Primary Health Care scores

Both caretakers and health professionals evaluated the quality of the PHC services provided to the children as unsatisfactory, as expressed by the general PHC score. If caretakers only consider the main attributes, the PHC services are considered to be adequate. If family- and community-oriented care is not considered, the health professionals’ score does not change regarding the quality of the PHC services: it is still inadequate.

#### Family- and community-oriented care

Although it is true that the FHS was not yet implemented in the town during the period of the study, this fact alone cannot explain these very low results. Most studies in Brazil show deficient family- and community-orientation, even if scores for these derived PHC attributes tend to be better in FHS units compared to traditional PHC units.^(^ ^20^ ^,^ ^23^ ^,^ ^24^ ^)^ Some authors tried to explain this by arguing that family orientation can be challenging in large urban centers or huge metropoles,^(^ ^25^ ^)^ however, this study suggests that even in rural areas, this orientation is lacking. Maybe part of the explanation is that few health professionals working in PHC services are trained in Family Medicine,^(^ ^26^ ^,^ ^27^ ^)^ and they have a traditional curative hospital-centered vision. For this reason, they treat the patients’ symptoms with medicines,^(^ ^28^ ^)^ but not integrate family or community aspects in patients’ management.

#### Degree of affiliation, use and first contact accessibility of the health center

Although the caregivers consider the health center as the entry point in the health system and use it very often, they rated the accessibility as low; this is in line with other studies in Brazil.^(^ ^20^ ^-^ ^24^ ^)^ However, it was the only attribute that the health professionals score as good. This can be explained by the low doctor density during the study period: 5.37 physicians per 10,000 children aged zero to 12; while the nurse density is 1.59 per 10,000 adult and child users. In a typical Brazilian small town, health professional density is much lower than the 23 health professionals (doctors, nurses and midwives) per 10,000 inhabitants appointed as adequate by the World Health Organization to provide essential maternal and child health care.^(^ ^29^ ^)^ Besides the number of professionals, organizational aspects such as a limited number of medical consultations may also be responsible for the low accessibility.

#### Long term person care

Long term person care was scored low by both caregivers and professionals. Literature shows different results on this attribute.^(^ ^20^ ^,^ ^21^ ^,^ ^23^ ^)^ In our study, this low score might be explained by the known high turnover of physicians delivering care for children in the town, as well as the fact that very few physicians are trained to provide long term person care.^(^ ^26^ ^,^ ^27^ ^)^

## Coordination of care: integrated services and information systems

The caretakers, in contrast with the professionals, consider the coordination of care as good quality; the scores for these attributes are higher compared with those mentioned in the literature.^(^ ^20^ ^,^ ^21^ ^,^ ^23^ ^)^

## Reflections of the feasibility of using Primary Care Assessment Tool-Brazil as a routine assessment tool

The evaluation of the quality of the PHC services provided to children in this small town shows that overall care is considered as inadequate with extremely low scores for family and community orientation.

Based on this study, it does not seem feasible to use the PCAT-Brazil as a routine assessment tool in this small rural town. Some recommendations were formulated from this experience: (i) foresee a budget to assess the quality of the PHC services provided to all users on a routine basis, in order to plan and evaluate PHC interventions; (ii) reduce the number of items of the long PCAT-Brazil assessment and validate short PHC assessment tools that can easily be used by healthcare managers; (iii) provide alternatives for classic paper versions of the PCAT-Brazil, such as machine-readable data forms that can automatically be validated and stored in databases available for analysis, or tablet-versions in which data can be stored on the device and transferred to a central database when a wireless connection is available; (iv) provide automatic data analysis platforms or free software programs in which conversions of obtained attribute scores can automatically be re-coded in scores between zero to 10, and general and essential PHC scores are calculated automatically; (v) gather data of all studies using PCAT-Brazil on one platform, allowing to compare obtained scores between municipalities or health regions; (vi) support municipal health professionals and managers with health care quality assessment, especially in remote rural areas, for instance by expanding telemedicine or other remote-platforms, in order to help planning PHC activities; (vii) strengthen the collaboration of medical educational institutions with remote PHC-services to facilitate such assessments; and (viii) expand Family Medicine training programs, including rural health internships, to enabling future medical specialists with adequate assessment tools.

PCAT-Brazil could be used on a routine basis and as a planning tool, particularly in a non-academic rural setting with the ultimate goal of providing good quality PHC-services for its users.

## CONCLUSION

This study provides insight on quality of provided Primary Health Care services in a small rural town in Brazil. We observed that there is room for improvement, especially concerning family and community orientation. The use of the PCAT-Brazil as a routine assessment tool seems not feasible in the given setting due to the high costs, lack of trained personnel and huge workload.
